# Ovarian Volume of Saudi Children and Adolescents in the Southern Region Based on Ultrasound Imaging

**DOI:** 10.7759/cureus.40147

**Published:** 2023-06-08

**Authors:** Sarah A Soliman, Mohammad A Algatheradi, Amal M Alqahtani, Abeer S Osluf, Nahid I Ali, Souad E Abbdellatif

**Affiliations:** 1 Diagnostic Radiology, Armed Forces Hospital - Southern Region, Abha, SAU; 2 Pediatric Diagnostic Radiology, Abha Maternity and Children Hospital, Abha, SAU; 3 Pediatric Endocrinology, Abha Maternity and Children Hospital, Abha, SAU; 4 Radiology, Armed Forces Hospital - Southern Region, Abha, SAU; 5 General Radiology, Abha Maternity and Children Hospital, Abha, SAU

**Keywords:** saudi arabia, chronology of age, endometrial thickening, uterine length, ovary volume

## Abstract

Introduction

Sonography is a non-invasive and painless technique used for assessing pelvic anatomy and disorder in children and adolescents. Ovarian growth patterns during infancy and puberty are not completely understood. No consensus exists about the normal measures and morphologic appearance of the ovaries in the southern region of Saudi Arabia. Therefore, this study determined the pattern of ovarian and uterine sizes among Saudi girls and their correlation with age.

Methods

This study was conducted in the radiology department at Abha Maternity and Children Hospital among girls between 0-13 years. All the participants underwent transabdominal ultrasound, and we measured ovarian volume, uterine length, and endometrial thickness to correlate with chronologic age using the Chi-squared test.

Results

A Total of 152 females were included in this study. The median age was 72 months, with a minimum of one month and a maximum of 156 months. The Chi-squared test showed a significant correlation between age and ovarian measurement. Age was positively associated with ovarian volume, uterine length, and endometrial thickness (p<0.001).

Conclusion

The study concluded that age strongly correlated with the size of the uterus and ovaries, which is crucial in interpreting ultrasound measurements of the pelvic organs correctly.

## Introduction

Measuring the volume of an organ is one of the most useful methods, especially in disease evaluation and diagnosing of many disorders [1,2]. In children, the knowledge of the developmental changes of the female reproductive organs is considered essential in investigating pelvic conditions in the early stages. Evaluating the volume of the ovaries in children and adolescents is an important factor in the diagnosis of ovarian disease and puberty-related disorders, and it is a useful indicator of the ovarian reserve [3,4].

Ultrasonography (US) measurement of the volume of the ovaries and uterus is considered one of the commonly used techniques in diagnosing puberty-related disorders [5]. It remains the most used and useful technique in pediatric and adolescent gynecology and is often the only one necessary before therapeutic intervention [6,7]. Since US is operator-dependent, high skills are required for accurate diagnosis. Though magnetic resonance imaging (MRI) is associated with better resolution than the US and is able to provide an excellent investigational modality for studies among girls with polycystic ovary syndrome [8], US is superior and cost-effective in evaluating female pelvic structures. It is also preferred because it is a non-invasive and painless technique that could be used for pelvic anatomy and different disorders in children and adolescents [9], as well as its availability in most hospitals and primary health care facilities. In contrast, MRI is required only when pathology is suspected or when the anatomy is not conventional (variant). Abdominal sonographic imagining requires an adequately distended urinary bladder as it displaces the bowel loops from the field, aiding in better transmission of sound waves and. thereby, the imaging [10]. Research showed that the mean ovarian volume increases from 0.7 ml in children at two years of age to 7.7 ml for 20-year-old adults and then declines through life to reach about 2.8 ml at menopause. The normal ovarian volume among premenopausal women is 5.3- 13.9 ml, while in those postmenopausal, the volume is lower than 8 ml [3,11].

The ovarian growth patterns during childhood are not completely understood, and there is no consensus about the normal measures and morphologic appearance of the ovaries among the pediatric age group [12]. Many studies consider ovarian volume; however, most of these studies were conducted among adult women, with limited reports on ovarian volume in children and adolescents. The main indications for pelvic ultrasonography in children and teenagers included early or late puberty, pelvic pain or tumor, ambiguous genitalia, primary amenorrhea, and vaginal bleeding in children. Little is known about the normal structure of ovaries in children because the prepubertal uterus is thin, while the uterine body is similar in size to the cervix. In addition, although cystic ovarian structures are also commonly observed in sonography, the classification of these structures is confusing and nonuniform [13]. Moreover, data on the ovarian volume in young girls is limited because of the lack of an easy and non-invasive method for accurately imaging the ovaries [3].

Our study aimed to determine the pattern of ovarian volume growth in girls from birth to 13 years of age in Southern Saudi Arabia and to identify the correlation between age and ovarian volume, uterine length, and endometrial thickness.

## Materials and methods

Study design, setting, and population

The study's design is a cross-sectional study conducted among girls between 0-13 years old referred to the radiology service at Abha Maternity and Children Hospital on March 20th, 2022.

Selected participants underwent transabdominal ultrasound for ovarian volume, uterine length, and endometrial thickness were measured to correlate with chronologic age.

Only female participants in the age range of 0 to 13 years old with Saudi nationality and who were referred to the radiology service at Abha Maternity and Children Hospital for abdominal discomfort were included in this study. Participants who are not Saudi, with an age range above 13, and with any congenital/surgical absence of the uterus or ovaries were excluded.

Sample size calculation

The sample size of the current study was estimated using the single proportion equation in the Raosoft package depending on a 95% confidence interval, and accepted margin of error accepted at 5%, and a response distribution of 90%. The calculated sample size was N=152 consecutive participants of girls after adding the dropout probability. The sample was randomly taken from eligible patients.

The data collection sheet included the following information; age, sonographic findings (ovarian volume, uterine length, and endometrial thickness), clinical information (known to have thyroid gland/adrenal gland abnormality, history of head trauma or congenital brain dysfunction, exogenous steroids, skin pigmentation/café-au-lait spots, and use of soy formula. We also recorded sonographic data readings.

Measurements of ovaries, uterus, and endometrium

**Figure 1:**
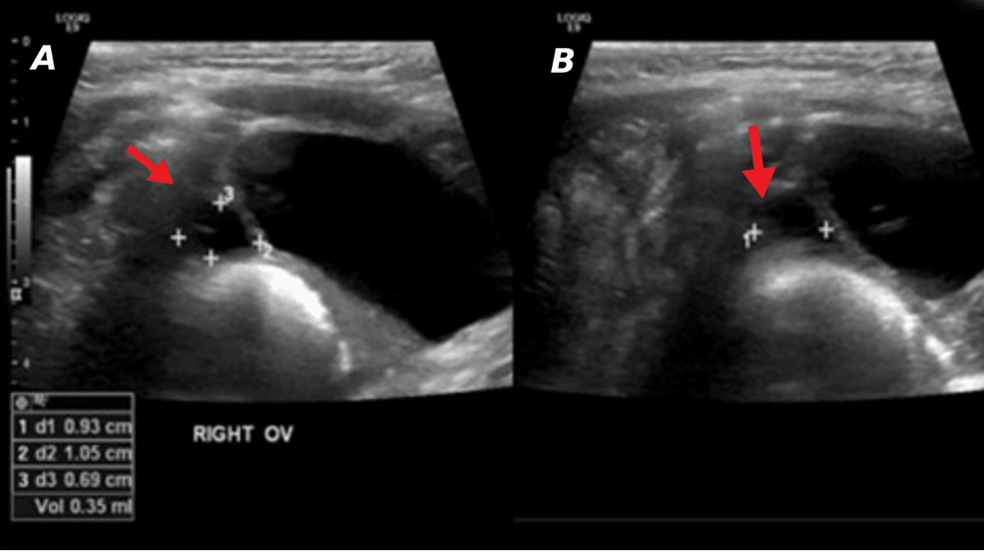
Normal ovaries (arrows) in a four-month-old girl A) Right ovary measures 0.3 ml with small follicles (transverse plane); B) Left ovary measures 0.4 ml with small follicles (longitudinal plane)

**Figure 2:**
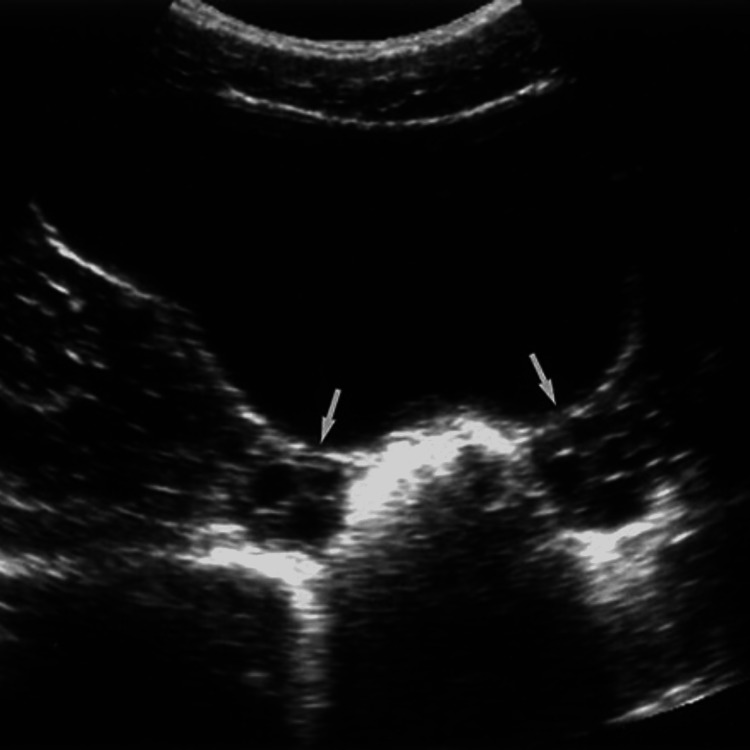
Effects of full urinary bladder during ultrasound scanning of clearly visualized the ovaries (arrows)

**Figure 3:**
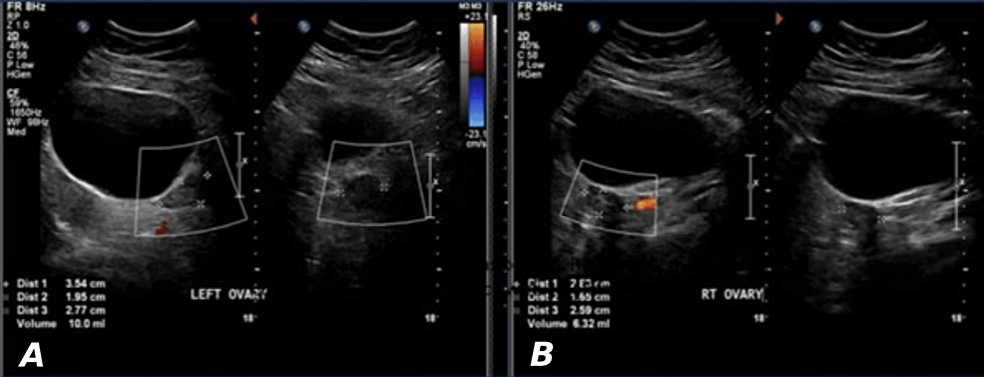
Normal ovaries in a 10-year-old girl with homogenous ovaries and few small follicles A) Right ovary measures 6.3 ml; B) Left ovary measures 10 ml

**Figure 4:**
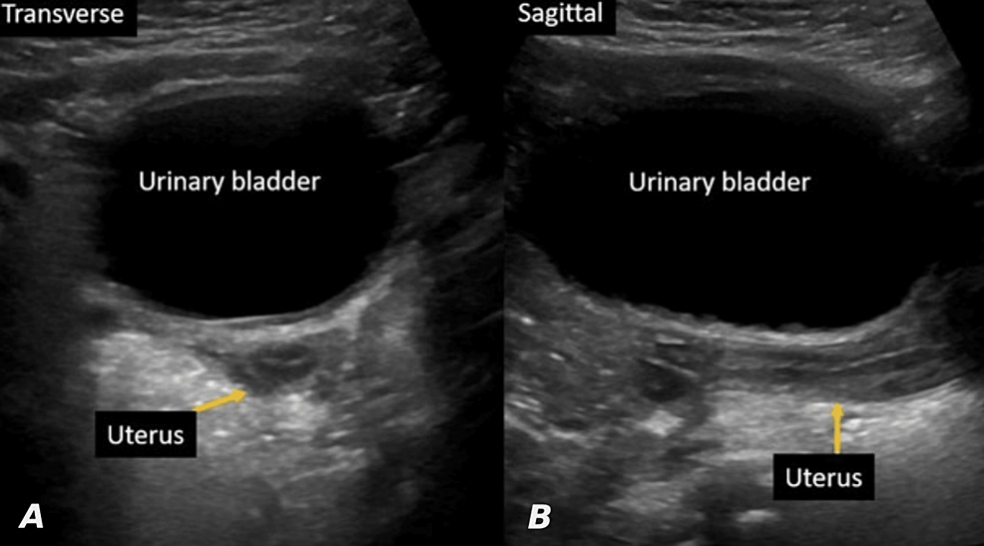
Transverse (A) and sagittal (B) images using the fluid-filled urinary bladders as an acoustic window showing a normal uterus in two-year-old girl

A mid-low frequency transducer (e.g., up to 5 MHz) and a full bladder were used as an acoustic window to achieve better imaging of the uterus and adnexa. The uterine length was measured in the midsagittal plane anteroposteriorly from outer-to-outer serosal surfaces. The ovaries were visualized as a homogeneous echotexture, and their volume on ultrasound was calculated by the formula: 0.523 × length (cm) × width (cm) × depth (cm) or automatically measured by the US machine and expressed in cubic centimeters (cm^3^). Figures 1-4 show sonographic scan images and measurements.

Data analysis

Statistical Package for Social Sciences software version 22.0 (IBM Inc., Armonk, New York) was used for data entry and analysis. Descriptive statistics were presented as numbers and percentages for categorical data and mean and standard deviation for continuous data. The Chi-squared test was used for the association between categorical variables. A p-value equal to or less than 0.05 was considered statistically significant.

Ethical consideration

The Institutional Review Board of the Abha Maternity and Children Hospital issued approval (H-06-091) for this study after explaining the idea of the research to the parent of the selected population and reassuring them regarding the tool of the study (ultrasound), which is non-ionizing radiation, harmless and painless tool. The parents consented for their children to be included in this research, and older children who understand the implications also assented to the participation. Confidentiality was assured, and no personal data (ID and names) of the selected populations were recorded. 

## Results

A total of 152 females were included in this study. The median age was 72 months, with an interquartile (IQR) range of 154 months. The minimum age in this study was one month, and the maximum was 156 months (13 years).

The mean right ovarian volume in ml on sonographic findings of all individuals was 2.09. The standard deviation was 2.57, the minimum right ovarian volume was 0.2, and the maximum was 17.00. The mean left ovarian volume in ml on sonographic findings of all individuals was 2.07. The standard deviation was 2.43, the minimum left ovarian volume in ml was 0.2, and the maximum was 17.00. The mean value of the uterine length in cm on sonographic findings of all individuals was 3.81. The standard deviation was 1.46, the minimum value of the uterine length was 2.00, and the maximum value was 8.7. The mean endometrial thickness in mm on sonographic findings was 2.94, the standard deviation was 1.81, and the minimum endometrial thickness was 0.1 to 10 (Table 1).

**Table 1:** Summary statistics (mean, standard deviation, maximum and minimum) of pelvic organ parameters

Variable	Mean (SD)	Min	Max
Age in months	6.74 (3.81)	0	156
Right ovary volume in ml	2.09 (2.57)	0.2	17
Left ovary volume in ml	2.07 (2.43)	0.2	17
Uterine length in cm	3.81 (1.46)	2	8.7
Endometrial thickness in mm	2.94 (1.81)	0.1	10

The comparison of the ovarian volumes, uterine length, and endometrial thickness among age groups showed a strong correlation between age and ovary structure. The increase in ovarian volume, uterine length, and endometrial thickness significantly correlated with increase in age (p<0.001; Table 2).

**Table 2:** Ultrasonographic measurements of the ovarian volume in milliliters, uterine length in centimeters, and the endometrial thickness in millimeters for the study participants per age categories p<0.001 indicates a strong correlation

Age categories (months)	N	Right ovarian volume (ml)	Left ovarian volume (ml)	Uterine length (cm)	Endometrial thickness (mm)
0-12	17	0.40 (2.5)	0.40 (1.80)	2.20 (6.70)	1.40 (5.00)
13-24	8	0.40 (0.20)	0.32 (0.40)	2.20 (0.70)	2.00 (0.50)
25-36	9	0.70 (2.22)	0.90 (2.20)	2.90 (1.00)	2.00 (1.00)
37-48	13	0.90 (9.70)	0.70 (9.70)	2.70 (1.00)	2.00 (2.00)
49-60	17	1.00 (2.00)	1.000 (1.60)	3.50 (2.00)	2.00 (7.00)
61-72	14	0.70 (2.50)	1.05 (4.60)	3.50 (1.30)	2.00 (2.90)
72-84	16	0.75 (4.0)	0.80 (10.40)	3.10 (2.10)	3.00 (1.00)
85-96	6	1.05 (2.30)	1.75 (7.30)	3.60 (1.00)	3.00 (1.00)
97-108	12	1.7500 (4.40)	1.90 (3.60)	4.00 (1.50)	3.00 (1.00)
109-120	7	1.30 (5.20)	1.80 (3.00)	4.00 (1.50)	3.00 (3.00)
121-132	6	4.1500 (3.30)	3.35 (3.90)	5.200 (2.80)	3.50 (2.00)
133-144	12	2.90 (10.20)	3.65 (6.60)	6.00 (3.50)	5.00 (9.10)
145-156	15	4.80 (15.00)	5.00 (15.00)	6.00 (3.10)	6.00 (8.30)
p-values		p<0.001	p<0.001	p<0.001	p<0.001

In this study, out of 152 females, only seven (4.6%) had thyroid gland/adrenal gland abnormalities, and 95.4% were normal. The frequency of head trauma or congenital brain dysfunction was in 17 (11.2%) individuals. The frequency of exogenous steroid use was 12 (7.9%). Skin pigmentation frequency was seen in four (2.6%) individuals. Soy formula was used by 17 (11.2%) individuals (Table 3).

**Table 3:** Ovarian abnormality-related morbidities

	Thyroid gland/adrenal gland abnormality		History of head trauma or congenital brain dysfunction		Exogenous steroid (estrogen-containing creams)		Skin pigmentation (cafe-au-lait-pigmentation)		Use of soy formula	
	N	%	N	%	N	%	N	%	N	%
Yes	7	4.6	17	11.2	12	7.9	4	2.6	17	11.2
No	145	95.4	135	88.8	140	92.1	148	97.4	135	88.8
Total	152	100	152	100	152	100	152	100	152	100

## Discussion

The purpose of this study was to evaluate the pattern of ovarian volumes, uterine length, and endometrial thickness and their correlations with age among Saudi children and adolescents.

We found a strong correlation between age and ovarian volume, uterine length, and endometrial thickness. There was no significant change in the volume of ovaries in the first 24 months of life, but after 24 months, we observed that the volume of the ovaries increased with age. This study's findings align with the study conducted by Asavoaie et al. [6] and other previous studies showing that an increase in age is directly associated with an increase in ovarian volume and uterine length [13,14]. Another study conducted in Turkey by Ersen et al. [15] found a statistically significant relationship between the increase in age with the increase in the uterine and ovarian volume in girls, supporting this study's finding showing a strong positive correlation between the volume of ovaries, the length of the uterus, and endometrial thickness and their age (p<0.001). This correlation is physiologically necessary to prepare the body for pubertal change and the transition to adulthood, with reproductive maturity [15].

Giligan et al. [16] conducted a study on 5647 females in the age range of 0 and 20 years and concluded that there was a correlation between the change in the volume of the uterus, ovaries, and uterine length and the menstrual cycle starting age. Our study showed similar results.

A study conducted in Korea in 2019 by Jin-Wook et al. on 180 females in the age range 0-18 years concluded that the age group of 10-12 years had the highest rate of increase in ovary size than other groups [5]. Their findings are similar to our findings, suggesting age-specific ovarian volume and size changes. Research showed that children with adrenal gland abnormalities had lower ovarian volumes than normal children of the same age, suggesting that adrenal gland abnormality is directly associated with decreased ovarian volume [17].

This study has some limitations to consider. The sample size was small and taken from only one hospital in the Southern region of Saudi Arabia, which might limit the generalization to all Saudi girls nationwide. We studied only age as an influencing factor, while both ovarian and uterine volumes can be influenced by multiple factors. We recommend further extensive longitudinal studies involving larger samples from all over the country to study other factors apart from age (i.e., socio-economic factors, climate change, etc.) to give more data that can be useful in child care, especially for Saudi girls.

## Conclusions

This study provided baseline data of normal measurements of the ovaries among healthy Saudi girls in the southern region that can help further research and diagnostics. The ovarian volume, uterine length, and endometrial thickness are significantly associated with age. This must be taken into account for accurate interpretation of US measurements of the pelvic organs in children and adolescents.

## References

[1] Gilja OH, Hausken T, Berstad A, Odegaard S (1999). Measurements of organ volume by ultrasonography. Proc Inst Mech Eng H.

[2] Bartlett JW, Frost C (2008). Reliability, repeatability and reproducibility: analysis of measurement errors in continuous variables. Ultrasound Obstet Gynecol.

[3] Kelsey TW, Dodwell SK, Wilkinson AG, Greve T, Andersen CY, Anderson RA, Wallace WH (2013). Ovarian volume throughout life: a validated normative model. PLoS One.

[4] Chen Y, Yang D, Li L, Chen X (2008). The role of ovarian volume as a diagnostic criterion for Chinese adolescents with polycystic ovary syndrome. J Pediatr Adolesc Gynecol.

[5] Jung JW, Yoo CH, Song KH, Choe BY (2019). Analysis of ovarian volume of Korean children and adolescents at magnetic resonance imaging. Pediatr Radiol.

[6] Asăvoaie C, Fufezan O, Coşarcă M (2014). Ovarian and uterine ultrasonography in pediatric patients. Pictorial essay. Med Ultrason.

[7] Ziereisen F, Guissard G, Damry N, Avni EF (2005). Sonographic imaging of the paediatric female pelvis. Eur Radiol.

[8] Yoo RY, Sirlin CB, Gottschalk M, Chang RJ (2005). Ovarian imaging by magnetic resonance in obese adolescent girls with polycystic ovary syndrome: a pilot study. Fertil Steril.

[9] Lippe BM, Sample WF (1978). Pelvic ultrasonography in pediatric and adolescent endocrine disorders. J Pedriatr.

[10] Garel L, Dubois J, Grignon A, Filiatrault D, Van Vliet G (2001). US of the pediatric female pelvis: a clinical perspective. Radiographics.

[11] Mohammad H, Ngwan SD, Utoo BT, Swende TZ (2013). Transvaginal ultrasound evaluation of ovarian volume among normal adults in Makurdi, North-Central Nigeria. J Reprod Biol Health.

[12] Orsini LF, Salardi S, Pilu G, Bovicelli L, Cacciari E (1984). Pelvic organs in premenarcheal girls: real-time ultrasonography. Radiology.

[13] Herter LD, Golendziner E, Flores JA, Becker E Jr, Spritzer PM (2002). Ovarian and uterine sonography in healthy girls between 1 and 13 years old: correlation of findings with age and pubertal status. AJR Am J Roentgenol.

[14] Naseem H, Javed AM, Aslam I (2015). Uterine Length and Ovarian Volume in Healthy girls of 1-13 years of age. Pakistan J Medical Health Sci.

[15] Ersen A, Onal H, Yildirim D, Adal E (2012). Ovarian and uterine ultrasonography and relation to puberty in healthy girls between 6 and 16 years in the Turkish population: a cross-sectional study. J Pediatr Endocrinol Metab.

[16] Gilligan LA, Trout AT, Schuster JG, Schwartz BI, Breech LL, Zhang B, Towbin AJ (2019). Normative values for ultrasound measurements of the female pelvic organs throughout childhood and adolescence. Pediatr Radiol.

[17] Vogt EC, Breivik L, Røyrvik EC, Grytaas M, Husebye ES, Øksnes M (2021). Primary ovarian insufficiency in women with Addison's disease. J Clin Endocrinol Metab.

